# Stem cell-derived mitochondria transplantation: a novel strategy and the challenges for the treatment of tissue injury

**DOI:** 10.1186/s13287-018-0832-2

**Published:** 2018-04-13

**Authors:** Jingyu Wang, Heyangzi Li, Ying Yao, Tengfei Zhao, Ying-ying Chen, Yue-liang Shen, Lin-lin Wang, Yongjian Zhu

**Affiliations:** 1 Department of Neurosurgery, The Second Affiliated Hospital of Zhejiang University School of Medicine, Hangzhou, 310009 China; 2 Department of Basic Medicine Sciences, School of Medicine, Zhejiang University, Hangzhou, 310058 China; 3 Department of Orthopedic Surgery, the Second Affiliated Hospital, School of Medicine, Zhejiang University, Hangzhou, 310009 China

**Keywords:** Mitochondria, Transfer, Transplantation, Mesenchymal stem cells, Injury

## Abstract

Damage of mitochondria in the initial period of tissue injury aggravates the severity of injury. Restoration of mitochondria dysfunction and mitochondrial-based therapeutics represent a potentially effective therapeutic strategy. Recently, mitochondrial transfer from stem cells has been demonstrated to play a significant role in rescuing injured tissues. The possible mechanisms of mitochondria released from stem cells, the pathways of mitochondria transfer between the donor stem cells and recipient cells, and the internalization of mitochondria into recipient cells are discussed. Moreover, a novel strategy for tissue injury based on the concept of stem cell-derived mitochondrial transplantation is pointed out, and the advantages and challenges are summarized.

## Background

Mitochondria as the center of cellular metabolism and production of energy arose from α-proteobacterium engulfed by a eukaryotic progenitor, consisting of inner and outer membranes that encapsulate the intermembrane space and matrix compartments [1]. In mammalian cells, mitochondria own their special circular and double-stranded genome, which is evolved from selective mitochondrial ancestor genes [2]. It is theorized that mitochondria in descendants are exclusively transmitted from their maternal individuals [3]. However, mitochondrial transfer between mammalian cells has been discovered that challenges the current inheritance of mitochondria and mitochondrial DNA (mtDNA), providing novel thinking for some diseases with mitochondrial damage or defect. The damage of mitochondria frequently occurs in the initial period of tissue injury. Release of accumulated ROS, imbalance of calcium ions, and insufficient supply of energy are responsible for mitochondrial damage, which aggravate cellular apoptosis and death around the injured region [4–8]. However, physiological properties of healthy mitochondria including replication, division, fusion, degradation, movement in the cytoplasm, and intercellular transfer provide the possibility of eliminating and replacing the damaged mitochondria [9, 10]. Previous published data show that injection of isolated viable respiration-competent myocardial mitochondria into the ischemic zone just before reperfusion would reverse postischemic functional deterioration and cellular apoptosis and limit infarct size [11]. Thus, replacement of damaged mitochondria may protect cells against further injury.

Stem cells including mesenchymal stem cells (MSCs), umbilical cord blood stem cells, embryonic stem cells, and induced pluripotent stem cells (IPSCs) with self-replication capabilities can differentiate into somatic cells varying in shape and function [12, 13]. Transplantation of stem cells recently became a hotspot of research to treat tissue injury. Meanwhile, MSCs originating from the mesoderm are favored in stem cell therapy for four reasons: the ability to maintain viability and regenerative capacity after preservation at −80 °C; simplicity of isolation and cryopreservation; the ability for rapid replication and high potential of multilineage differentiation; and minimal or even no immunoreactivity [14, 15]. Scientists are interested in the question of whether MSCs can transfer their mitochondria to somatic cells. The first report to reveal stem cells as donor cells of mitochondria is between MSCs and cancer cells with impaired mitochondrial respiratory function [16]. Cancer cells could accept healthy mitochondria from healthy cells to increase their invasiveness, tumorigenic potential, and resistance to chemotherapy [17–19]. Thus, inhibiting the process of mitochondrial transfer may play a vital role in cancer therapy.

Recently, numerous studies have shown that mitochondrial transfer from stem cells to injured cells has been considered a potential treatment for tissue injury. However, the specific mechanisms and critical factors remain to be identified. Consequently, in this mini review, based on the summary of the phenomena of mitochondrial transfer between stem cells and various mammalian cells, we present a novel strategy for tissue injury by stem cell-derived mitochondrial transplantation, and the advantages and challenges are summarized.

### Mitochondrial transfer in vitro

Material exchange represents a significant form of intercellular communication that permits not only transfer of small molecules or ions, but also transfer of identified intracellular structures including lysosomes, endosomal vesicles, plasma membrane components, and mitochondria in a unidirectional or bidirectional way [20, 21]. It has been revealed that mitochondrial transfer can restore the dysfunctional mitochondria in recipient cells, reprogram the differentiated cells, and even rescue the injured cells [22–24]. Spees et al. [25] first demonstrated that after coculture of A549 ρ° cells (lung adenocarcinoma A549 cell line with defective mtDNA) with MSCs, A549 ρ° cells could acquire functional mitochondria from MSCs. Also, the isolated mitochondria from the immortalized, untransformed mammary epithelial MCF-12A cells could easily enter malignant breast cancer cell lines such as MCF-7, MDA-MB-231, and NCI/ADR-Res cells rather than entering MSF-12A itself. After mitochondrial transfer, the proliferation of these cell lines would be suppressed in a dose-dependent pattern, and the sensitivity of MCF-7 cells to doxorubicin, abraxane, and carboplatin would be increased [26]. Intriguingly, vascular smooth muscle cells cocultured with MSCs induce upregulation of proliferation of MSCs through mitochondrial transfer [27].

Although it has been indicated that isolated mitochondria transfer to injured cells and replace the damaged or defective mitochondria to rescue function, the release of mitochondria may result in a series of immune responses. The mitochondrial components but not intact mitochondria are recognized as damage-associated molecular patterns (DAMPs), which induce strong proinflammatory reactions in the bloodstream and extracellular medium [28, 29]. For example, mtDNA released into extracellular space induces Toll-like receptor (TLR) 9-mediated inflammation and NRLP3-inflammasome activation [30, 31]. Collins et al. [32] injected mtDNA into mice joints, which results in inflammation and arthritis. Although the specific mechanisms remain unclear, adjusting the immune surveillance mechanisms of mtDNA and acquiring intact functional mitochondria may promote exogenous mitochondrial donation for therapeutic purposes.

### Mitochondrial transfer in vivo

Although the phenomenon of mitochondrial transfer in cell culture conditions has been widely observed, it is necessary to confirm whether mitochondrial transfer can occur in in-vivo conditions. Recently, evidence indicated that injured neurons are able to capture functional mitochondria from astrocytes [33]. CD38/CADPR/Ca^2+^ signaling may help astrocytes transfer mitochondria into neurons and promote survival and plasticity. In addition, Hayakawa et al. [34] collected extracellular mitochondria particles from primary mouse cortical astrocytes and then directly injected them into peri-infarct cortex of mouse models of focal cerebral ischemia. After 24 h, results suggested that transplanted functional mitochondria were indeed present in neurons and cell survival signals were amplified. Furthermore, Yi et al. [35] also observed mitochondrial transfer during embryonic development. They collected mitochondria concentrates from murine hepatocytes, and then injected them into zygotes from older mice. There were better developmental outcomes in the injected group than in the control group, which showed that mitochondrial transfer can improve embryonic development. Moreover, replacement of mitochondria through nuclear transfer among oocytes has turned into a research focus recently on the strategy for preventing the inheritance of mtDNA diseases [36].

Stem cells are recognized as unexceptionable donor cells for mitochondrial transfer and numerous studies have substantiated the significance of mitochondrial transfer in stem cell therapy, especially MSCs [37]. The first evidence for mitochondrial transfer as an in-vivo therapeutic tool came from Islam et al.’s study [38]. In the sepsis acute lung injury (ALI) model (airway-instilled *E. coli* LPS in anesthetized mice), bone marrow mesenchymal stem cells (BMSCs) transferred mitochondria to the alveolar epithelium that increased the generation of ATP and alveolar surfactant that regulates alveolar stability. In such nonsterile inflammatory diseases, alveolar macrophages activated by LPS that are widely used to mimic Gram-negative bacterial infection can accept mitochondria from stem cells to reduce the production of inflammatory factor while increasing the phagocytic ability and the production of ATP [38, 39]. Lung alveolar macrophages had been shown to gain mitochondria from MSCs in both in-vitro and in-vivo models of acute respiratory distress syndrome (ARDS) that result in an enhancement of macrophage phagocytosis activity and an improvement of bioenergetics, providing evidence for the therapeutic potential of mitochondria in acute, inflammatory lung disease [40]. In addition, in viral infection, Guo et al. [41] found that the formation of TNTs could be induced via porcine reproductive and respiratory syndrome virus between infected and uninfected cells, and mitochondria derived from stem cells transferred to infected cells depending on TNTs, which rescued infected cells from apoptosis/necrosis, whereas the mitochondria can be a vehicle to transport viral materials for spreading the infection.

In sterile inflammatory diseases induced via contusion, ischemia–reperfusion, or chemical injury, stem cells are capable of alleviating the inflammatory response and rescuing injured cells [42–44]. For instance, Naji et al. [45] indicated that the NLRP3–ASC–Caspase 1 axis induced via indium-tin-oxide nanoparticles in macrophages can provoke pyroptosis, while stem cells can inhibit the inflammatory process. In addition, MSCs rescue cardiomyoblasts from ischemia via direct cell-to-cell connections [46]. Li et al. [47] discovered that the devotion of mitochondria in MSCs provides great promise for the recovery of cigarette smoke (CS)-induced lung injury in chronic obstructive pulmonary disease. Meanwhile, it is reported that there is a higher mitochondrial transfer capacity in iPSC-MSCs than that from BMSCs to repair CS-induced mitochondrial damage. The reduction of the linear intercept value and the improvement in fibrosis were also greater in the group treated with iPSC-MSCs than in those treated with BMSCs [48]. Furthermore, mitochondrial transfer can also occur from MSCs to T cells in systemic lupus erythematosus patients.

Collectively, we summarize the latest studies of mitochondrial transfer via different kinds of stem cells (Table 1). Mitochondria from injured somatic cells are engulfed and degraded by stem cells, which results in induction of the cytoprotective enzyme heme oxygenase-1 (HO-1), and improvement of cellular proliferation and antiapoptotic function. Stem cells also donate their mitochondria to injured cells to resist oxidative stress and improve the state of cellular metabolism [49]. Thus, intercellular mitochondrial transfer holds a new approach to cure mitochondrial dysfunctional diseases using stem cells as a carrier [50].

**Table 1:** Mitochondrial transfer from different kinds of stem cells

Donor cells	Recipient cells	Defects	Methodologies	Effects	References
MSCs	A549 ρ° cells	Lack of functional mitochondria	In vitro: coculture	Rescue aerobic respiration	[25]
IPSCs MSCs	Airway epithelial cells	CS induced	In vitro: coculture In vivo: intravenous injection	Preservation of ATP levels	[48]
MSCs	T cells	Systemic lupus erythematosus	In vitro: coculture	Regulation of autophagy	[50]
MSCs	CECs	Rotenone-induced oxidative stress	In vitro: coculture	Mitochondrial function rescued	[68]
IPSCs MSCs	Myocardial cells	Anthracycline-induced damage	In vitro: coculture	Protection of damage	[85]
BMSCs	Alveolar epithelial cells	ALI	In vivo: airway instilled	Mitochondrial function rescued	[38]
BMSCs	Alveolar macrophages	ARDS	In vitro: coculture	Improvement of phagocytic capacity	[40]
BMSCs	H9c2 cells	Ischemia–reperfusion	In vitro: coculture	Reduction of apoptosis	[87]
BMSCs	Nucleus pulposus cells	Degenerative disc diseases	In vitro: coculture	Reduction of apoptosis	[88]
MSCs	Neurons	Ischemia–reperfusion	In vitro: coculture In vivo: intravenous injection	Reduction of brain lesion volume	[86]
MSCs	HUVECs	Ischemia–reperfusion	In vitro: coculture	Reduction of apoptosis and rescue of aerobic respiration	
BMSCs	Myocardial cells	None	In vitro: coculture	Reprogramming to the progenitor state	[22]
Endothelial progenitor cells	ECs	Adriamycin induced	In vivo: tail intravenous injection	Reduction of inflammation and apoptosis	[89]
MSCs	ECs	Asthma	In vitro: coculture In vivo: intravenous injection	Mitochondrial respiratory function rescued	

*ALI* acute lung injury, *ARDS* acute respiratory distress syndrome, *ATP* adenosine triphosphate, *BMSC* bone marrow mesenchymal stem cell, *CS* cigarette smoke, *EC* epithelial cell, *HUVEC* human umbilical vein endothelial cell, *IPSC* induced pluripotent stem cell, *MSC* stem cell including mesenchymal stem cell, *CECS* cornneal epithelial cells

### Mechanisms in mitochondrial release from stem cells

The first step of mitochondrial transfer is the release of mitochondria from donor cells. It has been suggested that mitochondrial Rho-GTPase 1 (Miro1) may be convenient for the release of mitochondrial transfer. Ahmad et al. [51] first suggested that Miro1 as a calcium-sensitive adaptor protein regulates intercellular mitochondrial movement from MSCs to epithelial cells (ECs). The authors developed an in-vitro system of coculture of MSCs and ECs as well as an in-vivo system of mice treated with MSCs via the trachea. The mitochondrial transfer was related to a remarkable recovery of impairment of mitochondrial function. Interestingly, mitochondrial transfer could be blocked when MSCs were preinduced with rotenone, a mitochondrial complex I inhibitor. They then examined the levels of mitochondrial intracellular transport-related proteins and suggested that only Miro1 was associated with the mitochondrial transfer. In addition, MSCs with stronger capacity of mitochondrial transfer than lung ECs and fibroblasts expressed high levels of Miro1 as compared to them. They further showed that, compared to control MSCs, the replacement of mitochondria from MSCs in which Miro1 was knocked down to injured ECs was reduced. This decrease was not due to the amount of TNTs, but the mitochondrial motility through the nanotubules. Other research showed that Miro1 protein plays a significant role in Ca^2+^ uptake into the mitochondria, which subsequently affects mitochondrial movement [52]. In conclusion, Miro1 is an integral protein involved in mitochondrial release from MSCs to ECs and Miro1-overexpressing MSCs are efficient mitochondrial donors with enhanced rescue potential.

At present there are three regulations for mitochondrial transport inside cells, which is believed to be involved in mitochondria release. The first is synaptic activity-dependent regulation. Mitochondria are transported to activated synapses in response to two intracellular signals that control their velocity and recruitment into the stationary pool. Identification of the KIF5–Milton–MIRO complex provides molecular targets to address this issue and studies independently identified MIRO as a Ca^2+^ sensor, providing a potential mechanism underlying Ca^2+^-dependent regulation of mitochondrial mobility [53–55]. The second regulation is neuronal signaling-mediated regulation. In dorsal root ganglion neurons, nerve growth factor (NGF) can act as a docking signal, causing axonal mitochondria to accumulate close to an external source of NGF [56]. Actin-based mechanisms appear to also have a role in this phenomenon. When neurons are treated with inhibitors of phosphoinositide 3-kinase (PI3K) or latrunculin B, an agent that destabilizes filamentous actin, mitochondria are not recruited to the NGF stimulation site, highlighting a crucial role for the PI3K signaling cascade in NGF-induced regulation of mitochondrial mobility. The third regulation of mitochondrial transport is related to microtubule-associated proteins (MAPs) [57]. Microtubules are dynamic structures and are stabilized by MAPs. Whereas MAP2 is specifically distributed in dendrites, MAP1B and tau are mainly axon-targeted MAPs. In addition to stabilizing axonal microtubules, tau has been shown to contribute to the regulation of the axonal transport of membrane organelles, including mitochondria [58]. Overexpressing tau in N2a and NB2a/d1 neuroblastoma cell lines, primary cortical neurons, and retinal ganglion neurons selectively inhibits kinesin-driven anterograde mitochondrial transport [58–60]. Recent studies have also revealed that the tau-mediated inhibition of axonal mitochondrial transport can be rescued by tau phosphorylation by MARK [61].

Moreover, the specific regulatory mechanisms of stem cell-derived mitochondrial transfer is still not clear. It has been suggested that the CD38/CADPR/Ca^2+^ signaling pathway mediates the mitochondrial transfer from astrocytes into neurons [33]. Thus, it is worth exploring whether this signaling pathway similarly works in stem cells. Are there any other factors and other signal pathways regulating the release of mitochondria? We speculated that the microenvironment of injured regions may send messengers to the stem cells to initiate the transfer of mitochondria. For example, whether molecules such as proinflammatory cytokines (IL-1, TNF-α) or anti-inflammatory cytokines (IL-4, IL-10), which are released in the early stage of injured tissue, can be recognized by stem cells and result in promoting or inhibiting mitochondrial release. It is interesting to know that dysfunctional mitochondria derived from injured cells can be engulfed and degraded by MSCs, which can result in induction of the cytoprotective enzyme heme oxygenase-1 (HO-1) and stimulation of mitochondrial biogenesis. As a result, stem cells are motivated and recruited, and then release their mitochondria to rescue the injured cells.

### Tunneling nanotube-dependent mitochondria internalization mechanism

Tunneling nanotubes (TNTs) are small membranous tubes with 50–1000 nm in diameter, which originated from stem cells during mitochondrial transfer. TNTs are thin cytoplasmic extensions bordered by a plasma membrane and connecting cells. TNTs were initially described by Rustom et al. [62] as a communicating intercellular transport network formed in coculture of human 293 cells and rat PC12 cells. Later, TNT formation was also reported in immune cells, including B, T, and NK cells, neutrophils, and monocytes, as well as in neurons, glia, cultured prostate cancer cells, and cardiac myocytes. TNTs seem to be a key point for effective mitochondrial transfer, inhibiting abrogating the transmission of cytoplasmic material such as mitochondria from BMSCs to epithelial cells [24], while inhibiting the process of endocytosis or phagocytosis shows little effect [63]. Onfelt et al. [64] observed that thin filaments involving F-actin and also a thicker subset (0.7 μm) containing both F-actin and microtubules participated in the formation of TNTs. Meanwhile, M-sec, a mammalian protein, can induce formation of TNTs that only contain actin filaments, but without microtubules [65]. In addition, exchange of cell particles between injured cells and stem cells was required for the formation of TNTs [66]. It also was observed that filopodial extension and retraction by stem cells draws an extension of TNTs from cardiomyocytes [67]. Also, Cdc42 (a small GTPase) plays a critical role in the TNT extension process [65]. Furthermore, mitochondrial transfer can be induced via mitochondrial damage that releases ROS to activate NF-κB and upregulate TNFαip2, enhancing the formation of TNTs [68].

The formation of TNTs was demonstrated to be controlled by some factors in in-vitro cultures, providing guidance for experimental settings and clues about how it might be regulated in vivo. It was observed that high concentrations of glucose play two-sided roles in the formation of TNTs. On the one hand, high concentrations of glucose diminished mitochondrial motility and inhibited mitochondrial trafficking in neurons via regulating Milton and its O-GlcNAcylation [69]. Moreover, TNT-mediated mitochondrial transfer from MSCs to endothelial cells was enhanced by glucose deprivation [66]. On the other hand, in other cell systems, high concentrations of glucose stimulated the formation of TNTs [70, 71]. In addition, it was reported that other factors including low serum, acidic conditions, H_2_O_2_ stimulation, viral infection, or use of chemotherapeutic agents promoted the formation of TNTs [70–75]. All of these studies revealed that the microenvironment around injured cells might be suited to the formation of TNTs, which is beneficial to the transfer of mitochondria.

### Microvesicle-dependent mitochondria internalization mechanism

Stem cells can also employ microvesicles (MVs) that range from 0.1 to 1 μm in diameter to transport their mitochondria to other cells. It has been reported that fitting mitochondria from MSCs were taken by arrestin domain-containing protein 1-mediated microvesicles that eventually were engulfed by macrophages [76]. At first, MSC-derived mitochondria in the cytoplasm were packaged into Autophagy Marker Light Chain 3-containing vesicles that then migrated to the cell periphery and were integrated into outward budding blebs in the plasma membrane. This MV-dependent mitochondria transfer was also reported between astrocytes and neurons [34].

### Gap junction-dependent mitochondria internalization mechanism

Live optical studies revealed that BMSCs formed Connexin43 (Cx43)-containing gap junctional channels with the alveolar epithelium, releasing mitochondria-containing microvesicles that were engulfed by the epithelium. In the sepsis ALI model, the distribution of Cx43 was uneven spatially in the alveolar epithelium, and BMSCs preferred to attach to the areas of high Cx43 expression. In addition, it was demonstrated that Cx43 was the critical connexin in the present gap junctional channel formation. Furthermore, BMSCs loaded with Ca^2+^ chelator successfully attached to the alveolar but failed to form nanotubes and microvesicles, which suggested that the Ca^2+^ was gap junctional channel dependent [38]. Subsequently, Cx43-based intercellular gap junctional communication also occurred in coculture of MSCs and endothelial cells [77]. These findings supported Cx43-dependent mechanisms and transfer of viable mitochondria in the protective response.

### Macropinocytosis-dependent mitochondria internalization mechanism

Macropinocytosis was suggested recently to play a role in mitochondrial internalization into cardiomyocytes [78, 79]. Ethyl isopropyl amiloride (EIPA) can abolish the therapeutic effect of mitochondrial transfer in h9c2 cardiomyoblasts as specific inhibitors of micropinocytosis [78]. To further assess the contribution of macropinocytosis in mitochondrial transfer, the inhibitory effects of other macropinocytosis and endocytosis inhibitors were measured by FACS after coincubation of EMCs with isolated DsRed2-labeled mitochondria. Mitochondrial transfer was reduced by cytochalasin D (inhibitor of actin polymerization) and nocodazole (inhibitor of microtubule assembly) but not by chlorpromazine (inhibitor of clathrin-mediated endocytosis), revealing that cells acquired exogenous mitochondria via micropinocytosis (not via clathrin-mediated endocytosis) [79]. In addition, integrity of the mitochondria outer membrane proteins and interaction with cellular heparan sulfate proteoglycan are essential for cells acquiring exogenous mitochondria.

### Actin-dependent mitochondria internalization mechanism

Studies have shown that internalization of mitochondria occurs through actin-dependent endocytosis and rescues cell function by increasing the ATP content and oxygen consumption rates. Some studies showed that mitochondrial internalization was not conducted by TNTs, caveola/clathrin-dependent endocytosis, and macropinocytosis. Only preincubation with cytochalasin D which has been well recognized to inhibit actin-dependent endocytosis and phagocytosis significantly decreased the internalization of mitochondria into cardiomyocytes and decreased the ATP content [80].

**Fig. 1:**
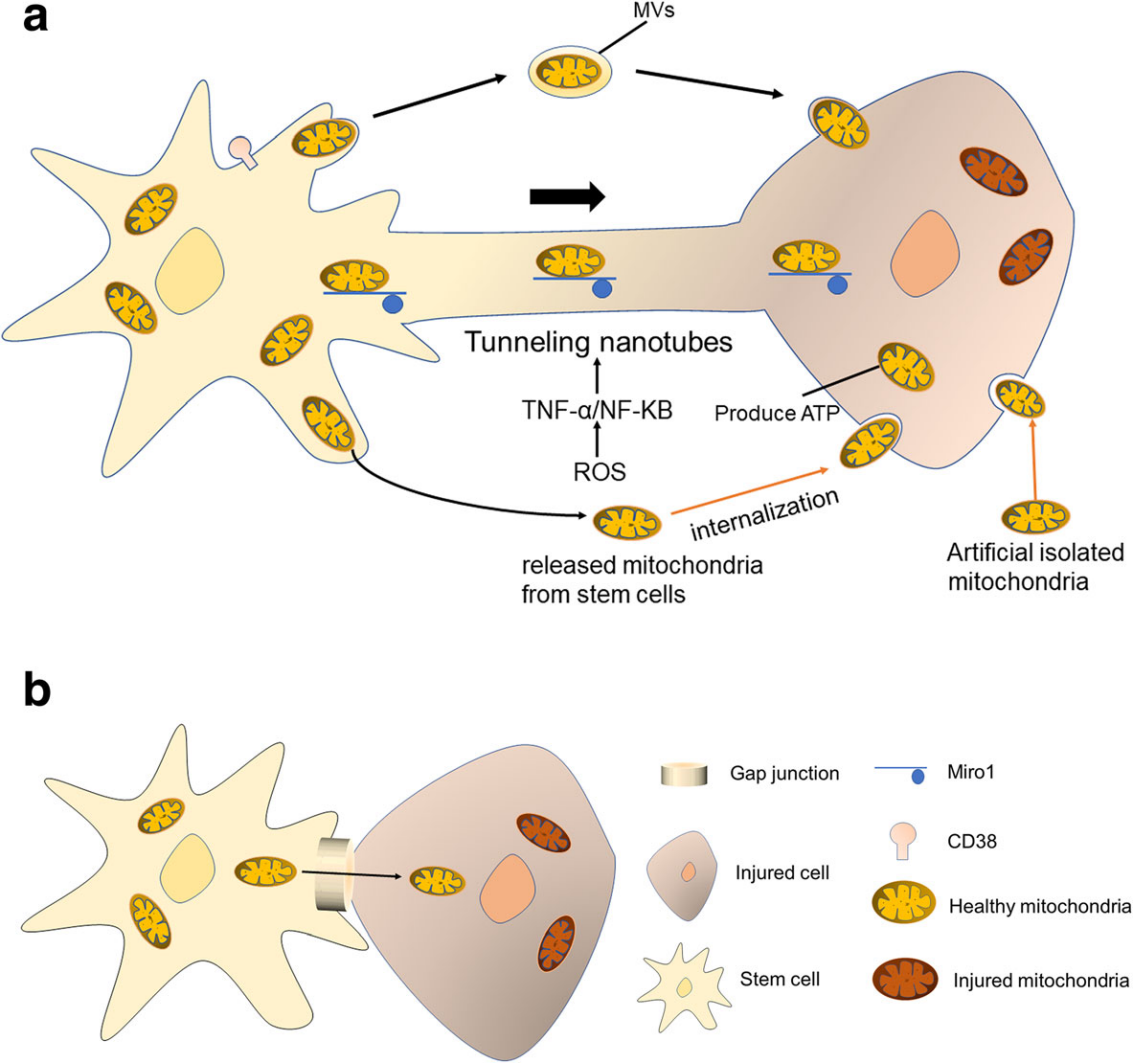
Mechanisms of mitochondrial transfer. **a** Formation of TNTs. Cells move apart and form TNTs with each other. Mitochondria can be transported in TNTs using Miro1 as a dynamic protein. Formation of TNTs can be stimulated via low serum, high glucose concentrations, OGD, or H_2_O_2_ that activate the ROS/TNF-α/NF-κB/TNFαIp2 pathway. Microvesicles (MVs) ranging from 0.1 to 1 μm containing mitochondria can be released from stem cells and engulfed by recipient cells. Mitochondria without MVs released from stem cells can be engulfed by recipient cells through micropinocytosis. Artificial isolated mitochondria can be engulfed by recipient cells through actin-dependent mitochondria internalization. **b** Gap junction-mediated mitochondrial transfer. Cells containing Cx43 proteins initially closely contact with target cells, followed by formation of gap junction. ATP adenosine triphosphate, Miro1 mitochondrial Rho-GTPase 1, NF nuclear factor, ROS reactive oxygen species, TNF tumor necrosis factor

The mechanisms of mitochondrial transfer are summarized in Fig. 1: Recipient cells passively phagocytose microvesicles containing mitochondria released by stem cells; tunneling nanotubes (TNTs), microtubules, or gap junctions occur between stem cells and donor cells for active replacement of intact functional mitochondria.

### Strategy and the challenges of stem cell-derived mitochondrial transplantation

**Fig. 2:**
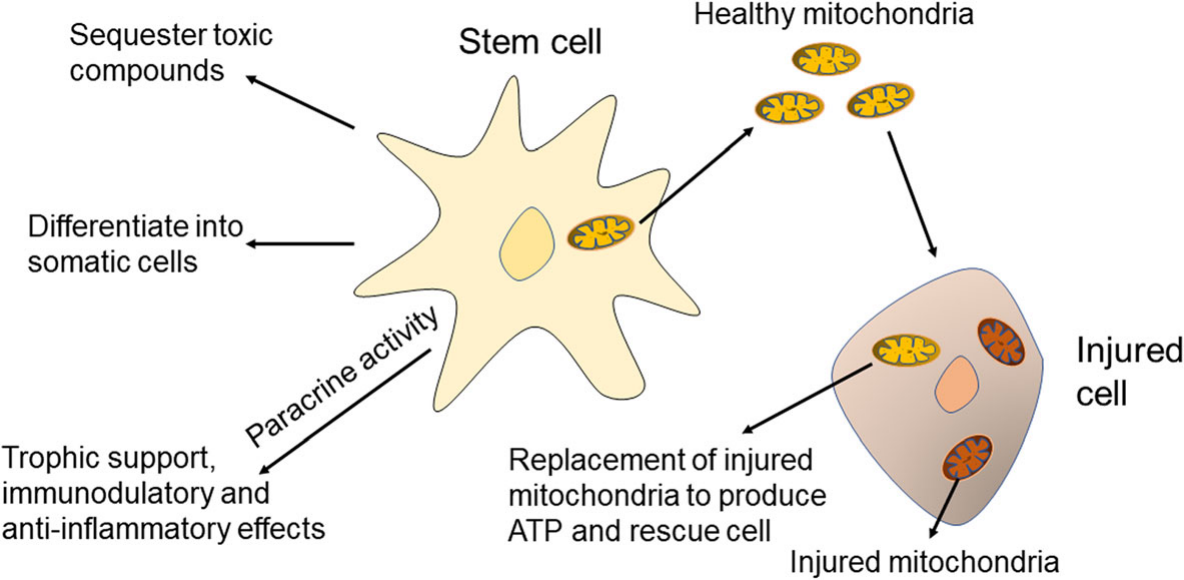
Mechanism of stem cell therapy. Stem cells became potential therapy for cell injury via various kinds of mechanisms. After cell injury, stem cells proliferate and migrate to damaged areas and then differentiate into degenerated somatic cells. Besides that, stem cells inhibit inflammatory effects that further damage nearby cells and remove toxic compounds accumulated via injured cells. In addition, growth factors that support cellular growth are released by stem cells. Importantly, healthy mitochondria derived from stem cells can transfer into injured cells to replace damaged mitochondria via TNTs or endocytosis. ATP adenosine triphosphate

Stem cells have been extensively demonstrated to rescue cell injury, with the specific mechanisms are summarized in Fig. 2. The mechanisms of stem cell therapy include the following aspects: differentiation into injured cells; sequester toxic compounds; paracrine activity for trophic support or anti-inflammatory effects; mitochondrial transfer by TNTs; and transfer of molecules via microvesicles [13, 81, 82]. Meanwhile, mitochondrial transfer between stem cells and injured cells becomes a novel mechanism, both for endogenous and exogenous stem cells. This raised the possibility to transplant stem cell-derived mitochondria to injured tissue as a novel strategy for stem cell-based therapy.

Stem cell-derived mitochondrial transplantation may have two main advantages. First, compared with MSCs, mitochondria possess the characteristic of lower immunogenicity because they lack the surface antigens on the surface membrane of MSCs. Thus, we raised the forward hypothesis that the injured recipient may accept stem cell-derived mitochondria from different individuals or even different species because of low immunogenicity. Second, stem cells have the ability for long-time proliferation and amplification, and can amplify to achieve the quantity of mitochondria which is required in clinical therapy.

However, there are many challenges for the use of stem cell-derived mitochondria for clinical treatment. For example, there are multiple technical questions to be resolved. First, how to isolate intact and viable mitochondria from stem cells? Although the intact functional mitochondria can replace the damaged or defective mitochondria to rescue cell function, mitochondrial DNA (mtDNA), but not intact mitochondria, results in a series of immune responses. Thus, to adjust the isolation technique to acquire high-quality active intact mitochondria may promote exogenous mitochondrial donation for therapeutic purposes. Second, how to keep the activity of mitochondria in vitro? Are cryopreserved mitochondria as effective as fresh isolated mitochondria? Third, how to meet the requirement of a sufficient quantity of mitochondria? Fourth, which approach of mitochondria transplantation into individuals should be selected from intravenous or local injection? Fifth, the development of specific fluorescence and mitochondrial tracking tools is required for further detecting the occurrence of mitochondrial transfer in vivo. Recently, there was inspirational discovery of a new tool called MitoCeption to track the transplanted mitochondria, providing a facility for research or application [83]. In addition, using transgenic mitochondrial labeling such as tGFP may be a viable option for tracking transplanted mitochondria both in vitro and in vivo, since the inner mitochondrial membrane can be labeled with high transfection efficiency and expression stability [84]. Finally, the consequence and security of mitochondrial transfer from different kinds of stem cells need to be further explored and evaluated.

## Conclusion

Mitochondrial dysfunction plays a vital role in tissue injury, revealing that restoring the function of mitochondria or replacement of damaged mitochondrial may improve cellular survival after injury. The reasonable application of mitochondrial transfer in mesenchymal stem cell-based therapy for sterile diseases such as myocardial ischemia–reperfusion injury, stroke, neuronal traumatic injury, or chemical nanoparticle-induced lung injury and for acute or chronic inflammatory diseases, such as ALI, will attract more attention in future [38, 48, 85, 86]. In these conditions, damaged cells are capable of capturing healthy mitochondria from stem cells to produce ATP, alleviate the inflammatory response, reduce apoptosis, and eventually rescue the injured cells [67, 86, 87]. We believe that this novel strategy for tissue injury based on the concept of stem cell-derived mitochondrial transplantation will be implemented soon in the near future if technical problems are resolved.

## Abbreviations

ARDS: Acute respiratory distress syndrome
CS: Cigarette smoke
EC: Epithelial cell
HO-1: Heme oxygenase-1
IPSC: Induced pluripotent stem cell
MAP: Microtubule-associated protein
Miro1: Mitochondrial Rho-GTPase 1
MSC: Stem cell including mesenchymal stem cell
mtDNA: Mitochondrial DNA
MV: Microvesicle
NGF: Nerve growth factor
PI3K: Phosphoinositide 3-kinase
TLR: Toll-like receptor
TNT: Tunneling nanotube
